# Genome Sequence of *Klebsiella oxytoca* M5al, a Promising Strain for Nitrogen Fixation and Chemical Production

**DOI:** 10.1128/genomeA.00074-12

**Published:** 2013-01-31

**Authors:** Guanhui Bao, Yanping Zhang, Chenyu Du, Zugen Chen, Yin Li, Zhu’an Cao, Yanhe Ma

**Affiliations:** a Institute of Microbiology, Chinese Academy of Sciences, Beijing, China; b University of Chinese Academy of Sciences, Beijing, China; c Department of Human Genetics, School of Medicine, University of California Los Angeles, Los Angeles, California, USA; d Tianjin Institute of Industrial Biotechnology, Chinese Academy of Sciences, Tianjin, China; e Department of Chemical Engineering, Tsinghua University, Beijing, China; f School of Biosciences, University of Nottingham, Nottingham, United Kingdom

## Abstract

*Klebsiella oxytoca* is an important microorganism for nitrogen fixation and chemical production. Here, we report an annotated draft genome of *K. oxytoca* strain M5al that contains 5,256 protein-coding genes and 95 structural RNAs, which provides a genetic basis for a better understanding of the physiology of this species.

## GENOME ANNOUNCEMENT

*Klebsiella oxytoca* is a Gram-negative bacterium that has shown promise in utilizing a wide variety of sugars to produce valuable chemicals and biofuels, including ethanol (1), hydrogen (2), 1,3-propanediol, and 2,3-butanediol (3). The species is also of agricultural importance due to its function in nitrogen fixation (4). To date, two complete genomes and seven draft genomes of *K. oxytoca* strains have been stored in the NCBI database, among which only strain KCTC 1686 showed the capability to produce chemicals (2,3-butanediol) (5). Compared with the other *K. oxytoca* strains, strain M5al does not have the regular polysaccharide capsule and is therefore more easily genetically accessed and modified. Moreover, the metabolic engineering of strain M5al for the production of 1,3-propanediol and 2,3-butanediol has been reported (6). Therefore, sequencing M5al will not only enrich the genome sequence database of *K. oxytoca*, but it will help to understand the genetic background of this useful strain. Here, we report the genome sequence of *K. oxytoca* M5al and describe the sequence differences from the genome of strain KCTC 1686.

The genome of *K. oxytoca* M5al was sequenced using the Solexa technology according to Illumina’s recommendations. A total of 6,199,416 reads, totaling 223,178,976 bases, were obtained, providing 37× coverage. Assembly was performed using AMOScmp (7) and Velvet (8), with comparative genome and *de novo* assemblies, resulting in an assembly of 282 contigs of >500 bp each. The final assembly includes 114 contigs after manual curation. The total size of the assembly was 5.8 Mbp, with an N_50_ of 94.98 Kbp and a G+C content of 60%. Compared to the 841-contig draft genome of *K. oxytoca* strain VJSK009, a mutant derived from the wild-type strain M5al (unpublished, http://genome.wustl.edu/genomes/view/klebsiella_oxytoca_m5al), our draft genome sequence has a significant improvement in assembly quality.

The genome sequence was annotated using the NCBI Prokaryotic Genomes Automatic Annotation Pipeline (PGAAP) (9). The draft genome sequence of M5al contains 5,351 genes, including 5,256 predicted coding sequences (CDSs), 19 rRNA genes, and 76 tRNA genes. A total of 4,450 orthologous genes between strains M5al and KCTC 1686 (GenBank accession no. NC_016612.1) were identified using the Reciprocal Smallest Distance (RSD) algorithm with the default parameters (10). These comparative analyses revealed 806 protein-coding genes that are present in strain M5al but absent in strain KCTC 1686. Among the 806 proteins unique to strain M5al, 231 proteins could be mapped into the Kyoto Encyclopedia of Genes and Genomes (KEGG) pathways, and most of these genes were related to purine, glyoxylate and dicarboxylate, methane, and nitrogen metabolism; this explains the genetic basis underpinning the function of strain M5al in nitrogen fixation and chemical production.

The genome sequence of *K. oxytoca* M5al serves as a basis for further investigation of the molecular basis of its potential in nitrogen fixation and chemical production. Relatively detailed annotations will reveal physiological difference among the various mutants derived from strain M5al, which showed differing capabilities to produce 1,3-propanediol (6).

### Nucleotide sequence accession numbers. 

This Whole Genome Shotgun project has been deposited at DDBJ/EMBL/GenBank under the accession no. AMPJ00000000. The version described in this article is the first version, AMPJ01000000.
